# Unlocking innovation: the dynamics of user engagement in an open-source 3D printer community

**DOI:** 10.1371/journal.pone.0321963

**Published:** 2025-04-16

**Authors:** Alysia Garmulewicz, Nicholas Sabin, Felix Reed-Tsochas

**Affiliations:** 1 Saïd Business School, University of Oxford, Oxford, United Kingdom; 2 Escuela de Negocios, Universidad Adolfo Ibáñez, Santiago, Chile; 3 Departamento de Administración, Facultad de Administración y Economía, Universidad de Santiago de Chile, Santiago, Chile; IIIT Kurnool: Indian Institute of Information Technology Designand Manufacturing Kurnool, INDIA

## Abstract

User innovation lies at the core of a growing body of research, with a predominant focus on how individual user attributes can be associated with observed contributions to innovation. However, framing user innovation in terms of individual attributes risks neglecting important changes in behavior at the individual and community level over time. Here, our focus is on how the time that individual users spend in that community relates to innovation outcomes. We study innovation in a firm-hosted online user community for a 3D printer company, marked by their commitment to open-source hardware. We analyze a unique longitudinal dataset of 38,277 observations recording user posts to an open-access user forum from October 2011 to March 2015. We find that time spent in a user community is positively associated with contributions to innovation, as users migrate from identifying problems and needs, to contributing innovation-related modifications and hacks. Our finding emphasizes the value of user retention in open-source communities as our analysis shows that typical users can add value to firms’ innovation activities over time. Our focus on open-hardware complements many existing studies on open-source software and advances our understanding of open innovation more generally.

## Introduction

The study of users’ contribution to the innovation process dates back to pioneering work in the 1970s and 1980s, when the then dominant idea of innovation taking place within the bounds of the producer firm was challenged by approaches that incorporated more complex innovation landscapes where users often took prime position [1–4]. In studying user innovation, a major focus of research has been to identify user attributes that lead to more valuable contributions to innovation [5]. Selecting and engaging with “lead users,” typically characterized by notable technical expertise or relevant knowledge, has proven to be a promising approach [3,5–9]. Studies have identified lead users according to their traits, status, and knowledge [7], and compared their contributions to innovation to internal lead users and typical users in terms of originality and utility of ideas [10]. The attributes of typical users have also been studied, finding that users’ knowledge of the underlying technology affects their propensity to contribute incremental versus radical ideas [11].

This study focuses on user innovation in the context of online firm-hosted user communities. While studies have looked at the role of firms in seeding knowledge in user communities [12], the role of employee-user interactions in the evolution of ideas [13], and how user interactions help develop and share knowledge over time [14], more empirical studies are needed for a deeper understanding of the temporal evolution of a range of potential behaviors in user communities. For example, although age in the community has been found to be negatively associated with the likelihood of contributing knowledge, on the other hand accumulating product experience over time has been positively associated with innovation [12,15]. This study allows us to quantitatively track user contributions to product innovation over time, and follow users migrating from identifying problems and needs, to contributing solution-oriented modifications and hacks. This is an important step in moving towards a more holistic approach that integrates the temporal evolution of user behavior with heterogeneous user attributes, so that the potentially unique behaviors of different groups within a community can be understood as they co-evolve.

A second key element of this work relates to the empirical context of studying the dynamics of an open-source hardware community. Despite conceptual outlines for expanding the empirical focus of open-source production beyond software [16,17], only a minority of studies have looked at innovation dynamics empirically in open-source physical product ecosystems [18,19]. The specific innovation dynamics in the context of open hardware, where skills, tools, and infrastructure differ from software, are under-explored [20]. The need to avoid generalizing across differing phenomena [21] underlines the importance of empirical study. By focusing on an open hardware ecosystem, this study contributes to a better understanding of the role that user innovation plays in the broader open innovation landscape beyond software [22].

We statistically analyze a unique longitudinal dataset of user innovation in the firm-hosted user community of Ultimaker, an open-source 3D printer [23]. 3D printing is a method of fabricating a physical object layer by layer, directed by a computerized design file. This makes 3D printing a type of digital fabrication, where digital information plays an active role in the fabrication of physical objects. Ultimaker makes open-source 3D printers where the core modules of hardware (physical machine), software (programs that run the machine), and firmware (embedded software for basic machine instructions) are developed by Ultimaker and associated developer communities, and freely revealed in the form of digital blueprints and source code. Our dataset of contributions to the user forum from 2011 to 2015 covers a period where Ultimaker arguably engaged in radical innovation. When the forum began, Ultimaker 1 was sold in a kit that had to be assembled by the user. In 2013 the Ultimaker 2 was released as an already-assembled integrated product. Whereas Ultimaker 1 was marketed towards hobbyists and educators, Ultimaker 2 was marketed towards businesses and industrial designers. In the words of Siert Wijnia, co-founder of Ultimaker, “because [Ultimaker 2] was not a kit anymore, and you sell a real product [Ultimaker 2] and it needs to work, then the whole game changed” [25]. Changes involved the development of new supply chains and extra measures of product safety compliance. The transition from Ultimaker 1 to Ultimaker 2 thus provides an example of a new product for a new market, an indication of radical innovation [26]. Ultimaker 2 enabled the company to become associated with professional desktop machines, a market it solidified with subsequent models. Moreover, the degree of new knowledge embedded in Ultimaker 2 encompassed not only the printer itself, but novel manufacturing and supply chain processes. This can be understood as radical innovation from the perspective of defining technology in terms of its knowledge components [27,28].

Since the company’s beginnings in 2011, Ultimaker has cultivated an active user community through a hosted online forum that has become a reputational hallmark. The community forum enables users to interact with other users as well as employees of Ultimaker. In this community we study the temporal evolution of user behavior. Our central research question is whether increased time in the community and comprehensive product engagement are associated with a users’ contribution to innovation. More particularly, we study users who hack or modify their 3D printer hardware, a type of innovation that has been shown to hold value for related firms [24]. To deepen the understanding of user behavior, we study user contribution to parts of the forum corresponding to different modules of the 3D printing system. This aims to capture breadth of understanding, or the level of comprehensive engagement with the product as it relates to contribution to innovation.

In studying user contributions to innovation over time, we find that time in the user community is indeed positively associated with contributions to innovation, thus supporting the idea that users can be of increasing value to companies over time. While there could be different underlying mechanisms that are consistent with this finding, such as increased technical expertise from a user’s professional development, we interpret this in accordance with the key studies on user innovation that link increasing product experience and needs awareness with contributions to innovation [15,29,30]. This is supported by our finding that users who have more comprehensive engagement with their product are more likely to contribute innovation. We find that this behavior is apparent and quantifiable at the community scale. More research is needed on differentiating potential drivers of innovative behavior, such as community versus individual learning effects.

Our findings suggest the importance of taking into account the potential of users to contribute innovation with time spent in a community. Understanding the empirical circumstances under which use-related knowledge can help or inhibit innovation adds an important layer of nuance. A prime implication is the importance of user retention in firm-hosted user communities within fast-moving tech sectors like 3D printing. In addition to identifying lead users, it is important to create meaningful and ongoing value to a broad base of users, as many of these may emerge as user-innovators given enough time and experience with their product. A second contribution of this study is insight into user innovation in a firm-hosted community for an open-source physical product. This is central given the differing challenges of producing hardware compared to software in an open-source environment [20]. Our study underlines the need to add greater nuance to the study of user innovation in open-source communities beyond software, considering a variety of organizational arrangements between firms, user communities, and user-producer communities that share a common interest in a products’ development.

The remainder of this article is organized as follows: as context for this work, we review the organizational literature on user and community innovation and open-source production. The central research questions and hypotheses are then outlined. The methods section begins with empirical background, providing illustrative examples of user innovation in the context of the Ultimaker user forum. This is followed by a description of the quantitative data, variable construction and model specification. To conclude, we present our results and discussion, limitations and future research.

## Literature review

### User and community innovation

Foundational studies in user innovation questioned the assumption that innovation takes place principally within the boundaries of a firm, and emphasized the importance of recognizing users as a valuable source of innovation [1–4]. A central insight in user innovation studies is the idea that innovation results from users having important information about their needs, and the transfer of this information to the producer, who may have the means to satisfy these needs through product innovation, is costly [31,32]. Information on a users’ needs and context of use is particularly difficult for a firm to replicate [32], especially when a user may not be fully aware of her needs when first using a product, as this awareness often results from repeated use of the product over time [33].

Over time, the field of user innovation has encompassed a great range of empirical studies, including the study of scientific instruments, industrial equipment, consumer goods, electronics [1,34–39] and services [40]. Here we focus on user innovation within online firm-hosted user communities, a locus of innovation that has emerged as the internet has decreased costs of firm-to-user and user-to-user communication [29,41–45]. User communities provide a means of governing innovation where participants in knowledge production are enrolled from outside of firm boundaries [22,46,47]. User communities have often formed in open-source product environments, most notably in software, but also in electronics and Internet of Things (IoT) [48]. Open-source software development communities are characterized by hybrid “user-producers” where innovations generated by users within the community are provided freely, allowing other users or a focal firm to benefit from their adoption and use [17,49].

Firm-hosted online user communities are designed to co-create value with users of their product [13]. User communities can be a source of rapid feedback on product development, and can be useful for monitoring, integrating, and receiving new ideas from distant users for innovation benefit [13,50]. This can be favorable to a firm due to new product features being shared between users, as well as new product innovations that the firm develops being sold on to users [43]. Firm-hosted user communities can also serve a range functions for various users and the host firm, from how-to guides and peer to peer troubleshooting and support communities, to modifications and suggestions of changes in the product architecture [51,52]. Efficacy of firm-hosted online user communities is associated with trust in the host firm [53] as well as perceptions of fairness [54], as tensions may exist if users feel their freely revealed innovations are subject to appropriation without due recognition or other forms of compensation. Willingness to participate in product innovation has been shown to be associated with characteristics of user creativity, and identification with and knowledge of the brand [55,56]. Organizational adoption of user innovation has been found to influence subsequent patterns of user innovation [57].

In user innovation within firm-hosted user communities, as well as user innovation more generally, a large focus has been on user attributes that lead to more valuable contributions to innovation [5]. There is good evidence that identifying and engaging with “lead users” is particularly useful [3,6]. Studies have identified lead users according to their traits, status, and knowledge [7,58], and compared their contributions to innovation to internal lead users and regular users in terms of originality and utility of ideas [5,8–10]. The attributes of ordinary users have been studied, finding that users’ knowledge of the underlying technology affects their propensity to contribute incremental versus radical ideas [11]. Position in the social network of peers in a user community, and being a professional (generating income from a product) versus amateur user have also been found to be important characteristics [59,60]. While traits and status focus more on intrinsic characteristics of personality and motivation, knowledge includes both usage experience, as well as more general knowledge about the product environment or relevant technologies [7,8,61]. This study compliments research on user attributes with a focus on the dynamics of user behavior over time.

### Open-source production

Here we review the literature on open-source production, emphasizing the study of open source physical goods to contextualize our empirical contribution. Open-source is a type of user innovation where those who contribute to a project expect to benefit from it as users, rather than for the purpose of selling [62,63]. Open source has become recognized as a novel form of organizing production, led in large part by the growth and increasing integration of open-source software into digital infrastructure. Emblematic projects such as GNU/Linux computer operating system and Apache server software have been joined by thousands of other projects who all adhere to the principles of open access, modification, and redistribution of the source code [16]. The persistence of open source for over more than 25 years as a viable mode of organizing production has led scholars of economics and organizational science to reevaluate core assumptions in the standard models of innovation and production [17,64], leading to new insights in innovation incentives, community dynamics, and product architecture, among others.

As the internet has enabled and accelerated new forms of collaboration and communication [42], the phenomenon of open-source production has emerged as a somewhat “startling” phenomenon given the free and open access nature of the good produced at private cost [65]. Scholars of organization science have tended to view open source as a new paradigm for organizing production [17,66]. Often associated with open-source goods, online communities are found to exemplify dynamics of knowledge collaboration where participants interact in ways that may benefit themselves as well as the overall community, such as engaging in collective problem solving [43,67]. These dynamics can take place without preexisting social relationships [67], and be sources of significant innovation [16,68]. Studies have further clarified questions related to structural enabling conditions [69], contributor incentives and motivations [32,66,70–73], community architecture [74–77], the effect of different licensing models and community size [63], and the subtleties of intellectual property rights [17,78].

While the overwhelming majority of open-source goods are software, here we focus on physical goods being produced by collaborative communities and released under open-source licenses [20]. Empirical examples include Arduino, an open-source electronics prototyping platform [79], DIY biology equipment [80], laboratory equipment including instruments for microscopy, molecular biology, and electrophysiology [81,82], and 3D printers [83,84]. One also sees open-source goods being used as platform technologies for the development of other goods, as in the use of Arduino and 3D printers to make open-source laboratory equipment [85]. While having physical components such as sensors and motors, most of these goods are examples of physical objects “entangled” with digital capabilities [86], with software being critical to the functioning of the product. While open-source hardware is clearly enabled by IT platforms, “sharing knowledge about atoms is not as frictionless as sharing bits” [20]. The fact that physical object design is an integral part of the product introduces new challenges for skills, tools, and infrastructure [20]. It is also important to note that in some circumstances, covering innovations under open source licenses is a cost that user innovators may not wish to invest in. This may be especially relevant in contexts where it is understood that users can modify designs without penalty.

Despite these potential frictions, it has been suggested that models of innovation for open-source environments could also apply to physical goods [16,17]. Unlike user innovation in open-source software, where users can freely distribute software through the internet, physical product innovation is expected to involve firm manufacturers in the production and physical distribution of products given significant economies of scale [16,87]. While open-source hardware introduces more challenges, it has been found nonetheless that open design processes for tangible objects can be organized to resemble open-source software development processes [88], primarily to publish documentation that supports community-driven product development, as well as to disseminate product innovations that were privately developed by a firm [20]. A number of studies have explored the behavior, success factors, and collective dynamics of open design participants on Thingiverse, a platform supporting the sharing of 3D printable designs [89–94]. Importantly, there is evidence of considerable variation in product and process openness in open-source hardware projects, with varying degrees of freedom to study, make, distribute, and modify. A main factor contributing to variation in openness is uneven quality of documentation of various complex components and manufacturing processes, including Computer Aided Design (CAD) files, assembly instructions, bill of materials, guidelines for participation, and associated licenses [96].

Open-source 3D printing companies and communities are part of this evolving and varied landscape. In general, open-source 3D printers have available the hardware designs for download and modification, and run on open-source software and firmware that similarly can be freely accessed, modified, and redistributed. In 2007 Adrian Bower and collaborators started the RepRap project with the vision of making a 3D printer that could replicate itself, and be open source for anyone to build, modify, and share. Since the first RepRap designs were released, a growing community has made, modified, and commercialized a large variety of 3D printers based on the initial blueprints, including proprietary models. For example, Makerbot is a company that initially based its product on an open-source RepRap design, then later removed their hardware, software, and firmware intellectual property from the public domain [89,95]. Ultimaker provides an example of an initial product design that was co-developed within the RepRap community, then evolved into being a private product where the designs remain open and freely disseminated among its user base. This study contributes empirically to the literature on open source production by exploring the dynamics of user innovation in the context of open source 3D printer hardware over time.

### Research questions and hypotheses

Within the field of user innovation and firm-based user communities, the study of how innovative behavior in user communities evolves over time has to date remained underexplored. Studies have looked at the role of firms in seeding knowledge in user communities [12], the interactions between employee and user idea contributions over time [13,47] and how users may develop and share knowledge over time [14]. While it appears clear that the particular characteristics and interactions within any given user community affect user contributions to innovation [14,51,62], more empirical studies are needed to understand the range of potential behaviors. For example, age in the community has been found to be negatively associated with the likelihood of contributing knowledge [12], while accumulating product experience over time has been found to be positively associated with innovation [15,29,30]. We seek to add clarity by studying whether time spent in the user community is positively associated with a users’ contribution to innovation in the Ultimaker user forum. As such our first hypothesis is:

1. H_1_: **Increased time in a user community increases the likelihood of a user contributing to innovation.**

To deepen our understanding of user behavior, we then study user engagement with parts of the forum corresponding to different modules of the 3D printing system over time. This aims to capture breadth of understanding related to a product. Product engagement and needs awareness have been linked to an increased likelihood to contribute to innovation, given that if a user understands his needs in different parts of the product system, there is a greater likelihood that that user can contribute to innovation that is of value for the firm [33]. With this in mind our second hypothesis is:

1. H_2_: **Comprehensive engagement with the product increases the likelihood of a user contributing to innovation.**

In exploring these two hypotheses, this study adds to the field of user innovation by complementing the strong focus on user attributes with a focus on behaviors of a user population over time. Taken together, these could lead towards a holistic approach that integrates the temporal evolution of user behavior with heterogeneous user attributes so that the potentially unique behaviors of different groups within a community can be understood over time.

Moreover, our contribution is in illuminating the dynamics of user innovation for an under-studied part of the open innovation landscape, that of open-source hardware communities. Informed by the classification of three types of openness in open hardware projects (the right to look, access and modify, or produce product designs) [96], we note that Ultimaker exhibits all three forms of openness. In releasing the hardware, software and firmware designs and code for their products, and asserting that the company is open to codevelopment by not claiming IP from other parties [97], Ultimaker offers an important empirical context for studying the contribution of users in an open innovation product ecosystem.

## Methods

### Ethics statement

This is a retrospective study of publicly available data posted by users to the Ultimaker forum between October 2011 to March 2015 [23]. The public data was accessed by the research team from March 18, 2015 to April 17, 2015. After the data was accessed, the research team de-identified the data by replacing user and thread identifiers with random numbers generated in R software. Statistical analysis was conducted using R software versions 2.14.1 to 3.1.2. Analysis and results only involve anonymized data that do not identify individual users. Given the public nature of the data and our adherence to ethical guidelines that protect individual privacy, our research did not require informed consent or Institutional Review Board (IRB) oversight. The collection and analysis methods complied with the terms and conditions for the source of the data, i.e. the Ultimaker Forum.

In the following sections we provide a qualitative overview of the empirical context of this study, providing illustrative examples of user contributions to innovation in the Ultimaker user forum. This helps to understand the nature of innovation in the user community and contextualize the quantitative study. Second, we introduce the quantitative data, including the structure of the user forum, and patterns of user contribution across parts of the forum. Third, we describe our variable construction. Fourth, we detail our model specification. These models enable us to study the evolution of behavior in the community over time, and distinguish between what are general changes in the community over time versus what is a result of an individual users’ time in the community.

### Empirical context: ultimaker user community

Ultimaker hosts a community forum where users of their 3D printers can voluntarily participate. The forum is used for multiple purposes, such as troubleshooting, assembly, fixing bugs in the software code, and sharing ways to modify the 3D printer. Employees of the company Ultimaker are also present on the forum and engage in discussion threads. All Ultimaker users have access to the source code of the 3D printer, including hardware blueprints, software and firmware code. On the forum in the hardware section, there is a category entitled “Modifications and Hacks” where users can post hacks or improvements to their individual machines. The production and distribution of new product releases is done by the company Ultimaker. To better understand user innovation in the context of the Ultimaker user community, we provide the following illustrative examples taken from the user forum category “Modifications and Hacks.” While other categories in the user forum are dedicated to building and operating the 3D printer, “Modifications and Hacks” allows users to post system alterations or new components that augment the printer designed by the company. These particular examples pertain to innovations in “Ultimaker 2,” the second released version of the Ultimaker 3D printer.

#### Feeder system.

The feeder system is the mechanism by which 3D printing filament, the material used, is fed into the nozzle of the printer for extrusion. The following posts are taken from a thread called “Ultimaker2 Feeder System - Improvements and Ideas” within the category Modifications and Hacks, where problems and alternative solutions to the Ultimaker feeder system are discussed. The thread begins by stating that the rear extruder has a design flaw, and then goes on to say:

The reason why I’m writing this thread is because I want to illustrate what I believe to be some of the main real reasons behind this problem and also organise efforts from me and other members here to design a true working solution. If we can design a really better solution to this problem and ultimaker adopts our efforts, then the ultimaker2 will become the first true BULLETPROOF 3D PRINTER!!

So lets pool our ideas and concepts, share files and models...and get a really hard nosed solution to this [] problem ... Ultimaker just added all the official source files for the extruder here!!

This post emphasizes the collaborative aims of the user, and their desire to improve the 3D printer. Rather than blaming Ultimaker for any perceived issues with the feeder system, the user frames it as an opportunity for collective user innovation. The availability of the source files, stated at the end of the post, is clearly important. In the posts that follow, a group of users begin diagnosing the problems in detail, and sharing design files for alternative feeder systems.

Later in the thread, a member of the company, responds:

One of the things that I am working on is improving the filament feeder. I read the posts in this thread to get more familiar with the problem. ... Thanks to all of you for all your feedback and ideas!

One of the possible solutions that we are working on is to eliminate the contact areas between the filament and the feeder housing. I printed a prototype of my redesign which we are going to test here at Ultimaker. However, I was wondering if some of you guys (who own an Ultimaker 2) would be interested in testing my redesign. If so, please let me know.

The Ultimaker company member further specifies the parts of their new design, and includes an image of the prototype. In a later post, after which alternative feeder designs have been proposed and tested by a number of users, the Ultimaker company member rejoins, stating that they are still following the thread, and that they have tried numerous options proposed by the users on the thread. The company member then continues with the following:

Unfortunately for the Ultimaker 2 we are going to have to act within the boundaries of the current tooling (injection molding dies are quite expensive tools). However I’ll definitely keep all the good ideas that I see on this forum in mind for a future feeder design.

The new machines are now fitted with a revised version of the feeder housing...Besides that we have performed some research in order to get more insight into the resistance induced by the various elements in the extrusion train. I’ve written a test report about the method that we used and about the first results. If anyone would like to read this report: let me know and I’ll make sure that you’ll receive a copy.

This exchange clearly illustrates the role that the forum plays for Ultimaker as a company, both in gathering new ideas from the community, and interacting with users as testers for new solutions. The explicit sharing of the new designs, manufacturing constraints, and plans for the future also illustrates the transparent relationship between users and the company when it comes to innovation. Overall, the exchanges on this thread illustrate how contributions of the users are of direct value to the improvement of the product as viewed by the host firm.

#### Heater block.

A second example illustrates a user modification that while not directly absorbed into the core R&D process of Ultimaker, came to be an “add-on” that users can buy from the company. This is a heater block, a part of the extruder of the 3D printer that heats the filament as it passes through and out the nozzle. An Ultimaker user invented a new, more robust heater block in order to print boron carbide filament. Not being designed for such hard materials, the nozzle on the original heater block wore away quickly. The user developed a new design that allowed new nozzles to be replaced easily, and posted it on the Ultimaker forum:

Hi! I have printed some very abrasive home-made filament lately which eats my expensive Ultimaker 2 nozzle a bit too quickly...So lately I have been putting some effort in finding a better design. Here is the result:

Among other users, a member of the Ultimaker R&D department responded to the post, thanking the user for his input, clarifying the features of their design, and asking the user questions in terms of its functionality.

A group of users began to adopt the heater block that allowed nozzles to be easily interchangeable, finding an additional use in enabling experimentation with a variety of materials that required different nozzle diameters. Over time, these users organized the production of small batch orders. The new heater block is now available from numerous Ultimaker printer re-sellers, and Ultimaker provides guides for installation as an official “hardware upgrade” for the Ultimaker 2 model. The new heater block is an example of user innovation that increased the functionality of the 3D printer for users. Recognizing this, Ultimaker promoted it as an extension to their product.

The larger dataset on user contributions to modifications and hacks encompasses both core and peripheral user innovation [98]. The importance of both is evident in the feeder system and heater block innovations. Core innovations are provided by a principle contributor, as in the case of the heater block, or a subset of users in the case of feeder system. Other users contributed peripheral innovations by testing their designs, asking questions and offering feedback and in some cases, changes to their initial designs. These users play a key role in validating the designs to a certain extent, while also providing important information to the host firm monitoring the thread.

While members of the company Ultimaker are seen to participate actively in certain innovation-related forum threads, this is not always the case. Sometimes users share modifications that are of high use-value but there is no apparent involvement from the company. An example is the multiple threads on designing a heated bed, the platform upon which material is deposited. A heated bed can improve print quality by avoiding warping by keeping the deposited material warm. While later generations of the Ultimaker included a heated the bed, the first generation did not. This led users to propose designs and query others on the forum whether there was any interest in a kit. Interestingly, Ultimaker later offered a heated bed kit as an add-on that could be bought. Thus, while there isn’t direct evidence of the Ultimaker team interacting with users on this subject, this user need was addressed by the company and integrated into later designs of their product.

There are also examples of user innovations that are not adopted by Ultimaker as a company, nor many other users. For instance, a thread on enclosures that seal the 3D printer and help keep heat in featured a variety of enclosure solutions, including a top enclosure and even a heatproof bag that covered the entire printer. While this provoked some interested responses, there is no evidence of such designs being integrated into later models, or offered as add-ons. Rather, side panels were integrated into later models, and a more modest front panel enclosure offered as a model add-on. While not all proposed user innovations are adopted by the host firm Ultimaker, there are many instances where the innovation is of added value to the users themselves. Participants in the Ultimaker forum may gain from finding and applying innovations from other users, as well as receiving feedback on innovations proposed by themselves. In each case, the forum provides a medium for users to increase the utility and functionality of their product.

### Description of the quantitative data

Our dataset comes from the open access Ultimaker user forum, where each forum post, associated to a unique user ID, was recorded between October 2011 and March 2015 [23]. October 2011 was when the forum was first founded. After the data was collected, the forum was substantially reorganized, meaning that a longer period of data collection would have resulted in inconsistent data. As such, we interpret the online behavior of users in the specific context of the time period that the data was collected. At the time of data collection there were a total of 2,423 unique user IDs in the forum. Although it is reasonable to assume that all users on the forum are users of an Ultimaker 3D printer, being a member in the forum is not an obligatory part of owning an Ultimaker 3D printer. There is no fee to join, and the forum is open to search for non-members, so anyone without a forum membership can browse questions and answers on the forum. Thus the forum should not be taken as representative of the total number of users of Ultimaker 3D printers; rather, the forum reflects a community of users who are interested in contributing their time and interacting with other users of Ultimaker 3D printers. While not necessarily representative, this community is nonetheless engaging with a representative set of user topics and concerns related to using a 3D printer, from basic topics such as how to set up the machine, to more advanced topics such as machine modifications. Given data is sourced only from within the user community, we cannot control for the effects of external events on user behavior.

The architecture of the community forum is organized according to the basic architecture of the 3D printing system, with hardware, software, and firmware as the main sections of the forum. Within the sections there are forum categories, and within these are threads. Each post is part of a specific thread, and therefore nested within this overall structure. While forum categories are set by Ultimaker, users can add new threads and contribute posts. Modules, categories, threads, and posts are mutually exclusive, meaning that a post can only be in one thread, a thread can only be in one category, and a category can only be in one module. Table 1 summarizes the structure of the Ultimaker user forum. As the focus of our analysis, the category Modifications and Hacks is in the Hardware module and focuses on innovations related to the product. Other categories of the forum, such as Assembly, Troubleshooting, and Cura (the software that programs the 3D printer) focus on knowledge sharing and user assistance in setting up and running hardware, software, and firmware modules of the 3D printer. While the main modules of the 3D printing system are common to many products with digital and physical architecture, the organization of categories and threads is particular to the Ultimaker user forum, a caution to generalizability.

**Table 1:** Ultimaker community forum structure.

Modules	Hardware	Software	Firmware
Categories (total = 12)	1. Assembly	1. Replicator-G	1. 5D
	2. Troubleshooting	2. CAD	2. Sprinter
	3. Modifications and Hacks	3. Other	3. Alternatives
		4. Cura	4. Marlin
		5. NetFabb	
Thread number (total = 4,301)	2,164	1,944	193
Post number (total = 38,277)	22,014	14,665	1,598

Notes: as of March 2015, there were 2,423 unique user IDs in the forum. The user forum is structured according to the main modules of a 3D printer: hardware, software, and firmware. Each module has categories of threads related to subjects within these modules. The total number of threads (4,301) is shared across hardware (2,164), software (1,944), and firmware (193). Each thread is made up of individual posts by users. Hardware has 22,014 posts, software has 14,665, and firmware has 1,598 for a total of 38,277 posts in the user forum. The category Modifications and Hacks is in the Hardware module and contains 9244 posts, or 24% of the total number of posts in the user forum. Other categories of the forum focus on knowledge sharing and user assistance in setting up and running hardware, software, and firmware modules of the 3D printer.

**Fig 1:**
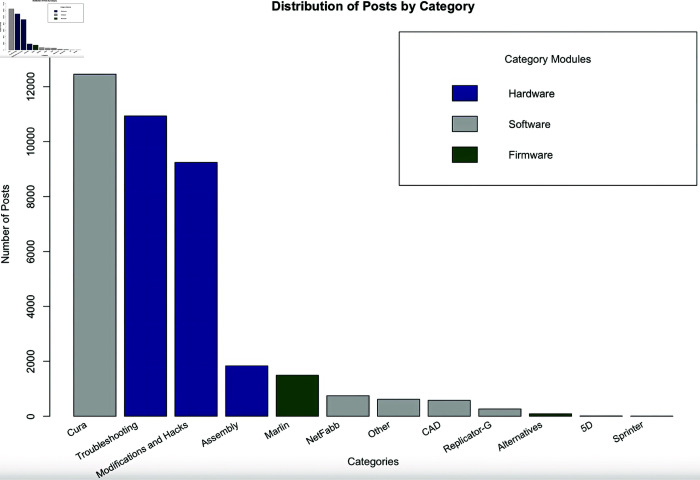
Distribution of posts by category. The number of posts per category of the user forum is shown, with colors signifying whether each category is part of the hardware, software, or firmware modules. “Cura” (the software that runs the 3D printer), “Troubleshooting” (helping users set up and use their 3D printer), and “Modifications and Hacks” (adaptations and contributions to product innovation) are the most active parts of the user forum.

Fig 1 illustrates the number of posts per category in the forum. “Cura,” “Troubleshooting,” and “Modifications and Hacks” are the most active parts of the user forum, where Cura refers to the core software that runs the Ultimaker 3D printer, Troubleshooting allows users to get guidance and help in setting up their printer and using it, and Modifications and Hacks is associated with user contributions to product innovation. Modifications and Hacks as a thread category has 24% of the total number of posts in the forum, constituting a major area of forum usage.

**Fig 2:**
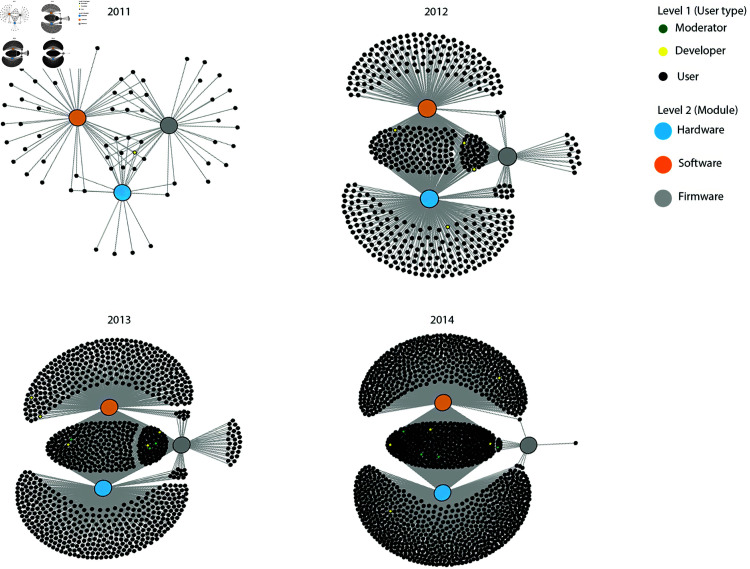
User contributions by module. User contributions to different modules of the user forum (hardware, software, and firmware) are visualized using a bipartite network graph. Level 1 is the user, level 2 the module, and a link signifies a user posting at least once during the time window. Patterns are visible between users who post in one module (periphery), and users who post in multiple modules (core). Developers and moderators are demarcated, and are located in the core signifying posts across multiple modules.

One can also visualize user contributions to different modules of the user forum. An analysis was done to understand patterns of user activity in yearly time frames by modules in the forum associated with the main architecture of the 3D printer. Each post is confined to a single module, but a user can post across modules. This data structure of users potentially engaging across multiple modules can be well-represented with a bipartite network. Fig 2 applies a bipartite network approach to representing the data, where level 1 is the user, and level 2 the module [99]. A link between a user and a module signifies that the user was posting at least once in that module during the time period observed. This approach lets one visualize to what extent users post in single modules versus posting in multiple modules. Yellow and white nodes signify developers and moderators, respectively. In the graphs one can clearly distinguish between users who post in one module (in the periphery of the graphs), from users who post in two or three modules (at the core of the graphs).

As expected, moderators and developers are clustered in the core of the network graphs, signifying a tendency to post in two or three modules. Yet there is a substantial number of users also in the core. We see that the number of new users in the periphery and the core grows over time, signifying increasing numbers of users who post in one as well as more than one module in the user community. This core-periphery pattern remains relatively stable over time. We also conducted the analysis with monthly and weekly time frames, with the substantive results being the same. What is not evident is whether users change their position in the graph over time, migrating from the periphery to the core. To formally test this, we proceed to the statistical model specification.

In order to test the hypotheses in this study it is necessary to clearly distinguish between changes that are happening in the community over time from changes in user behavior resulting from each individual’s time in the community. The longitudinal trends explored here are incorporated into our model specifications. An active user signifies a user who posts at least once in the time frame observed. In parallel, the number of posts in the user forum also increased over time.

**Fig 3:**
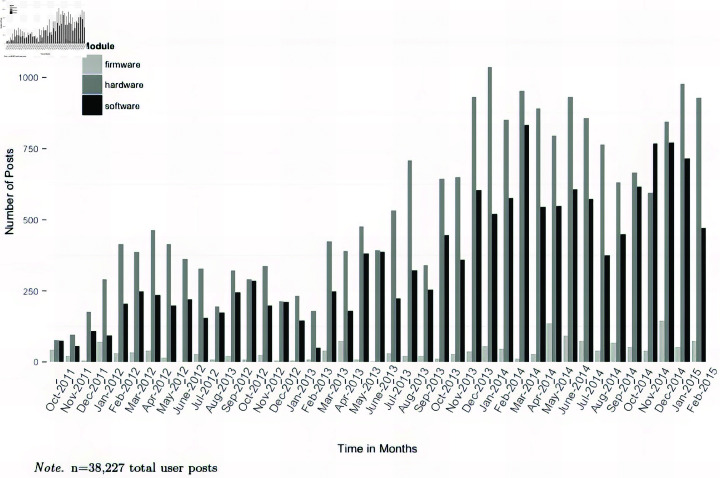
Number of posts by module. The number of posts by module (hardware, software, and firmware) is visualized over time in the user forum. One can see a substantial increase over time for the number of posts.

Fig 3 illustrates the number of posts by module in the forum over time. One can observe from this figure that posts in hardware and software increase over time. Posting behavior in the firmware area of the forum is much lower. This is reflected on in the discussion section.

### Variable construction

In this section we statistically test the core hypotheses developed from the literature. These concern two different behaviors. This first is how time spent in the community relates to innovation. The second is how comprehensive product engagement relates to innovation. Table 2 provides descriptive statistics for the variables used. Independent variables are age in the community and comprehensive/non-comprehensive product engagement. Control variables are calendar time, activity level of users, and whether a user is a moderator or developer. The dependent variable is contribution to innovation.

**Table 2:** Descriptive statistics: users in the ultimaker community forum 2011-2015.

Total user posts	38,277				
Level 1: User observations (monthly)	5,814				
Level 2: Unique user IDs	2,423				
Variables	N	Mean	SD	Min.	Max.
*Independent variables*					
Age in the community (scaled and centred)	5,814	.00	1.00	–.65	4.89
Age in the community (in months)	5,814	4.72	7.22	.00	40.00
Comprehensive/non-comprehensive engagement	5,814	.23	.42	.00	1.00
*Control variables*					
Calendar time (scaled and centred)	5,814	.00	1.00	–2.30	1.35
Calendar time (in months)	5,814	26.17	10.95	1.00	41.00
Activity level of users (scaled and centred)	5,814	.00	1.00	–.38	16.80
Activity level of users (unscaled)	5,814	6.58	14.67	1.00	253.00
Moderator/Developer (coded 1 for Moderator or Developer; 0 otherwise)	2,423	.04	.19	.00	1.00
*Dependent variable*					
Innovation contributors/non-contributors	5,814	.35	.48	.00	1.00

Notes: The data is structured with two levels, with level 1 being user posts within a monthly time interval, and level 2 being unique user IDs. Independent variables are age in the community and comprehensive/non-comprehensive product engagement. Control variables are calendar time, activity level of users, and whether a user is a moderator/developer. Contribution to innovation is the dependent variable. Variables are centered and scaled for computational reasons of model convergence. 38,277 user posts are aggregated as 5,814 observations of users’ monthly behavior. For the statistical models we use two levels of data. Level 1 is monthly measure of innovation for each user, and level 2 is the unique ID of each user.

#### Dependent variable.

The dependent variable in the analysis is a binary variable, with 1 denoting a user who contributes innovation, and 0 denoting a user who does not. Contribution to innovation is measured by posting activity to threads in the category “Modifications and Hacks,” where users can post and discuss their own or others’ system alterations. The dependent variable is constructed per monthly interval of age in the community, enabling the tracking of user behavior over time. Users must post at least once to the category “Modifications and Hacks” to be counted; the threshold of 1 was chosen given any single post could be of value concerning product innovation. While posts to the user forum can be understood as constituting a spectrum of innovation and utility, creating a more fine-grained categorization of posts on the basis of their value to innovation was empirically impractical, given the subjective nature of how useful a particular post is for other users and the Ultimaker company. For example, contributing a new product idea may be either a significant contribution, or irrelevant depending on its utility. Even a failed idea could be useful for other users, signaling that a direction for further product modification is not useful. Similarly, while agreeing or questioning a thread on a product innovation may appear to carry lesser weight than a post proposing a modification, such assurances or queries could be the key to the modification’s success. As such, contributions to innovation as conceptualized as inclusive of core and peripheral user innovation [32]. That is, a post within the category “Modifications and Hacks” may contain a significant new product prototype or modification, or rather may contain supporting information for the refinement of other user innovations [32]. Here we adopt both types of contributions as valid instances of user innovation.

#### Independent variables.

To study the first hypothesis, that of increased time in the user community being positively associated to to a users’ contribution to innovation, we use age in the community as a measure for time spent in the community. Age in the community is thus the independent variable of interest, constructed by measuring each users’ age in the community at monthly intervals, starting from each users’ respective join date. Age in the community ranges from 0 to 40 months, meaning the maximum number of months a user has spent in the Ultimaker forum is 40, and the minimum 0. In the models to follow, the variable for age in the community is centered and scaled for computational reasons related to model convergence.

To study the second hypothesis, whether comprehensive product engagement leads to contributions to innovation, a measure of whether each user is non-comprehensive or comprehensive during each monthly age interval is added as an independent variable. This is constructed as a binary variable, with 1 denoting a user who posts across more than one section related to the system architecture (hardware, software and firmware), and 0 denoting a user who posts in only one section, per monthly interval of age in the community. This approach is related to the study of user contributions by system architecture [100]. If a user posts in multiple sections within one month, they are classified as comprehensive, and if a user posts in only one section during a monthly time period, they are classified as non-comprehensive. The results were checked for robustness to the time interval chosen by running the models with time measured in weeks. As this did not significantly affect the results of the model, months were chosen being less sensitive to a low post number per time interval. Given the average number of posts for a user within a monthly time frame is 39, one can reasonably classify a user who is posting every time in the same section within a month as non-comprehensive, and in different sections within this time frame as comprehensive. The use of a monthly age interval in the community enables the tracking of change in user behavior over time.

#### Control variables.

Calendar time, user activity level, and the status of a user as a moderator/developer versus general user are included as control variables. Calendar time is constructed as a continuous variable in monthly intervals. As discussed in the above section on longitudinal trends, calendar time is added in order to clearly distinguish between changes that are happening in the community over time from changes in user behavior resulting from each individuals’ time spent in the community. Including calendar time in the models also controls for unobserved events in the forum that may affect user behavior. Activity level, an important measure in online communities [101] is a measure of the number of posts a given user makes per monthly age interval in the community and controls for the effect of users who post infrequently as well as high posting users, that is, the heterogeneity of intrinsic activity levels for different users. Activity level also controls for unobserved socialization. The status of a user as either a moderator/developer is as binary variable, coded 1 if the user is designated as a moderator or developer in the forum, and 0 if the user is not. Developers are employed by Ultimaker, and thus have different incentives in contributing to the forum. Moderators are designated by the community and appear to be users that have high levels of contribution and are recognized for their expertise [44]. The effect of both moderators and developers is controlled for in order to focus the analysis on typical user behavior. In all models, calendar time and activity level are centered and scaled for computational reasons related to model convergence.

### Model specification

A key concern for studying both hypotheses is the need to distinguish between the longitudinal trends occurring in the user community versus changes occurring in individual behavior over time. A regression-based approach can statistically test our hypotheses and accommodate the distinction between longitudinal community trends and individual behavior. To address this we use a hierarchical generalized linear model for repeated binary response data [102]. Model choice is motivated by the multilevel nature of the data, as users often make multiple posts and should not be treated independently. More specifically, a logistic model for binary data is appropriate because the dependent user behavior in each time period is coded as 0 or 1 as described above. In all models, random intercepts are included for each user ID to account for interdependence of observations. Level one of the model constitutes observations of users per age interval in the community, totaling 5,814 observations. Level two of the model constitutes unique user IDs, totaling 2,423. We thus model 5,814 observations of 2,423 unique users in the Ultimaker forum over the period of 2011-2015. A logistic random intercept model is estimated, expressed in the following form:


$$\begin{array}{ccc} logit(P_{ij})=\gamma_{0}+\overset{r}{\underset{h=1}{\sum }}\gamma_{h}x_{ij}+U_{0j} & & \end{array}$$ (1)

The model expresses the log-odds, or logit of $P_{ij}$, as a sum of a linear function of explanatory variables. $P_{ij}$ is the probability of contributing innovation for time window *i* and user *j*. In all models, $\gamma_{0}$ denotes the model intercept; $x_{ij}$ denotes explanatory and control variables for each time window *i* for user *j*, summed to *r* for the number of variables in the model. *γ*_*h*_ denotes the coefficient fit for each variable *h* to *r*. We now proceed with fitting the model to the empirical data.

## Results

Table 3 summarizes the results of modeling the effect of age in the Ultimaker community on the likelihood of contributing innovation, as well as the relationship between comprehensive product engagement and contribution to innovation. The results are organized to first provide a baseline control (Model 1) before introducing each independent variable (Models 2 and 3). The final model (Model 4) combines all variables of interest with the complete controls. All models were tested for potentially influential observations (using Cook’s distance) or problematic multicollinearity of the variables. Neither of these issues were found to significantly affect the results.

Regarding the empirical results, we see in Model 1 that the activity level of users is a significant control (coefficient estimate of 2.153 and p-value < .001) and continues to be significant across all models. The variable for moderator/developer is marginally significant in Model 1 (p < .05) but does not remain significant in Models 2-4 when the key explanatory variables are introduced. Calendar time is not statistically significant in Model 1, but is significant in later models (Model 2: p-value < .001 and Model 4: p-value < .01) when the variable for age in the community is included. This suggests that the variables for age in the community and calendar time are capturing distinct temporal mechanisms as suggested in the variable descriptions.

**Table 3:** Hierarchical logistic regression predicting the effect of age in the Ultimaker community and comprehensive product engagement on the likelihood of contributing innovation, 2011-2015.

	Model 1		Model 2		Model 3		Model 4	
	Coefficient	S.E.	Coefficient	S.E.	Coefficient	S.E.	Coefficient	S.E.
Age in the community	–	–	0.402***	(0.065)	–	–	0.399***	(0.063)
Comprehensive engagement	–	–	–	–	1.56***	(0.130)	1.550***	(0.129)
Calendar time control	–0.025	(0.059)	–0.291***	(0.073)	0.023	(0.056)	–0.214**	(0.068)
Activity level of users	2.153***	(0.154)	2.160***	(0.148)	1.321***	(0.144)	1.351***	(0.144)
Moderator/Developer	2.223*	(1.005)	1.681	(0.905)	1.544	(0.851)	1.109	(0.822)
*Constant*	–2.269***	(0.302)	–1.877***	(0.196)	–2.303***	(0.176)	–2.071***	(0.154)
*Deviance*	5682.5		5644.6		5533.5		5493.3	

Note: This table provides estimates from hierarchical generalized linear models for repeated binary response data, estimating the likelihood of contributing innovation. The dependent variable across all models is a binary measure of user contribution to innovation in a given time period (1 denoting contribution). Regressors are age in the community and comprehensive engagement with the product. All regressions control for calendar time, activity level of users, and whether the user is a moderator/developer. Model 1 is the baseline control model. Model 2 tests for the effect of age in the community on the likelihood of contributing innovation, model 3 tests for the effect of comprehensive product engagement on contributing innovation, and model 4 includes both explanatory variables. In all models, random intercepts are included for each user ID. *N*=5,814 monthly observations nested in 2,423 unique user IDs. Standard errors are in parentheses. *p < .05, **p < .01, ***p < .001 (two-tailed tests).

Recall that Hypothesis 1 regards the effect of time spent in the user community on contribution to innovation. In order to judge the empirical support for Hypotheses 1, we focus on the results from Models 2 and 4 because they include the variable for age in the community. We see that age in the community has a positive coefficient and significant p-value in both models (Model 2: coefficient estimate 0.402, p-value < .001; Model 4: coefficient estimate 0.399, p-value < .001). This provides empirical support for the first hypothesis that increased time spent in the community is positively associated with a user’s contribution to innovation.

**Fig 4:**
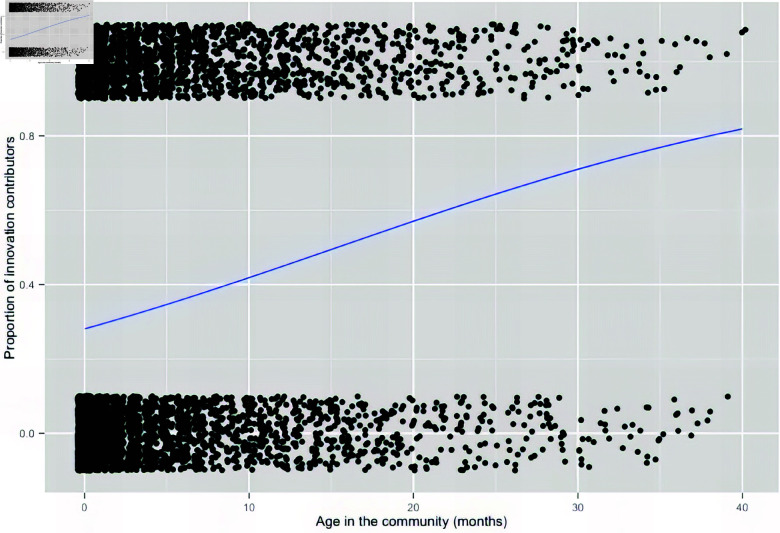
Likelihood of contributing to innovation according to age in the community. The relationship between contribution to innovation and age in the community is visualized by fitting a logistic curve to the data, with data points representing a measure of a user as either a contributor (1) or non-contributor (0) to innovation. We can observe a positive slope to the curve, indicating that the proportion of users who are contributing innovation rises over time with age in the community.

These results are also presented visually in Fig 4, where each data point represents a measure of a user as either a contributor or non-contributor to innovation per monthly age interval in the forum. Vertical jitter is added to the data points around 0 and 1 to make the results more interpretable. The figure helps us visualize the relative density of data points, as contributor or non-contributor, as the user ages in the community. The positive relationship is evidenced by fitting a logistic curve to the likelihood of contributing to innovation according to age in the community.

Recall that Hypothesis 2 regards the effect of comprehensive engagement on contribution to innovation. To judge the empirical support for Hypothesis 2, we focus on the results from Models 3 and 4 as they include the variable for comprehensive engagement. We see that comprehensive product engagement has a positive coefficient and significant p-value in both models (Model 3: coefficient estimate 1.560, p-value < .001; Model 4: coefficient estimate 1.550, p-value < .001). This provides empirical support for the second hypothesis, that users who engage more comprehensively with their product by posting across sections of the user forum associated with different parts of the 3D printing system, are more likely to also contribute to innovation.

Model 4 allows us to view the effect of both explanatory variables in the same model. Model 4 indicates that both age in the community and comprehensive product engagement have a positive relationship with contribution to innovation and are both positive at a p-value of < .001. This suggests these variables are capturing distinct aspects of a user’s product knowledge. It is also evident in Model 4 that being a moderator or developer does not appear to relate to the likelihood of contributing innovation. This clarifies that users, rather than firm employees or designated moderators, are the primary contributors to innovation in the Ultimaker user forum.

## Discussion

The first finding of this study is that the more time a user spends in the user community, the more likely they are to contribute to innovation. This supports hypothesis one. This means that users, inclusive of typical and lead users, can be viewed as potential sources of innovation that firms may want to cultivate over time through a hosted user community. This finding for the user community can be consistent with a range of different underlying mechanisms that we can associate with individual users, such as users accumulating knowledge from their profession or learning about related technology [15,29,30], or being influenced by their peers in the community [59]. A particularly compelling mechanism at the individual level is that as users spend more time in the user community, and their experience with the product increases, their understanding of their own user needs also increases. Greater product engagement and needs awareness have been linked to an increased likelihood of contributions to innovation [33]. This accords with the support found for hypothesis two, that more comprehensive product engagement leads to contributions to innovation. Comprehensive product engagement, measured by looking at the extent to which a user is interacting with multiple components of their 3D printer, is found to be positively associated with contributions to innovation. This suggests that a more holistic, or integrated understanding of a product leads to a greater likelihood of making contributions to innovation. As such, this study adds further support for the idea that accumulated product experience over time is positively associated with innovation [12,15]. These findings offer a theoretical contribution to the study of user innovation by complementing the focus on user attributes with the behavior of a user community over time. Our study thus provides an important step in moving towards a more holistic approach that integrates the temporal evolution of user behavior with heterogeneous user attributes. Nonetheless, when interpreting the data one should be aware of the limitations of assuming causality in the observed relationships.

At a practical level our findings suggest the importance of accounting for the potential of users to contribute innovation with time spent in a community. Developing a comprehensive understanding of how use-related knowledge may help or inhibit innovation is critical to avoiding the risk of overlooking certain segments of the user community as potential sources of innovation. While identifying lead users with specific knowledge and capabilities has innovation benefits [3,6], so too does ensuring users remain engaged in a community over time. A prime implication is the importance of user retention in firm-hosted user communities. It is important to create meaningful and ongoing value to a broad base of users, as many of these may emerge as user-innovators given enough time and experience with their product. This may be particularly pertinent to open-source products, where the right to freely modify and adapt products for user needs is enjoyed by users. In facilitating users to engage with a host firm over time, the design of the community forum is paramount. Providing a dedicated section for modifications and hacks encourages users to engage in this subject, channeling community interactions inclusive of those with the host firm on product innovation. While providing a platform for community interaction on product innovation, it also enables the host firm to more easily capture useful contributions to innovation.

A further contribution of this research is based on the empirical setting itself, where user innovation is studied as it relates to an open-source physical product. By studying the dynamics of an open-source hardware community, this work contributes to a better understanding of the role that user innovation plays in the broader open innovation landscape beyond software [22]. Our study complements the study of communities sharing 3D printable designs [89–94] with a focus on user innovation related to 3D printer hardware and engagement across hardware, software, and firmware modules. More specifically, our study provides insight into the phenomenon of users beginning their engagement with a product by first focusing on areas like machine set-up and troubleshooting, and over time evolving their engagement to focus on contributions to hardware innovation. In the case of Ultimaker, this is facilitated by access to hardware blueprints as well as software and firmware source code. This is significant given that most studies of user innovation in an open-source environment have focused on software, with the majority of studies of physical goods being limited to those that are proprietary in nature. While the dynamics between users and manufacturers are similar to traditional examples of user innovation, given that the manufacturer produces and sells the good, and the user modifies or hacks it, the users have access to the source code and blueprints of the entire product architecture, and are actively contributing modifications at that level. The contributions of user innovators in this product environment thus constitute a boundary case between user innovation in traditional physical goods and open-source software. Our study underlines the need to add greater nuance to the study of user innovation in open-source communities, considering a variety of organizational arrangements between firms, a user communities, and user-producer communities that share a common interest in a products’ development.

### Future research

When using observational data of user contributions over time it would be beneficial to have additional controls, such as user experience or expertise prior to their participation in the user forum. This would strengthen the interpretation of the results in this study, as well as allow the integration of user traits and characteristics with the temporal evolution of user behavior. This would enable one to study patterns of contributions to innovation over time for different user types or characteristics, perhaps uncovering different temporal signatures. More specifically, an important research question is the extent to which the characteristics of users, such as their traits, status, and knowledge [7], drive contributions to innovation in a user forum over time. Further study is also suggested to directly test the theoretical mechanisms underlying the positive relationship found between time spent in the user community and contributions to innovation. A more causal interpretation of the data could be enabled by randomizing the explanatory variables of interest. This would clarify whether experience with the product is an important mechanism as suggested. With this further understanding, firms could be more purposeful in how they cultivate and engage with different types of users over time, with benefits both to the firm and the user community. An important area is the consideration of potential feedback loops between user contributions to innovation, firm adoption, and user engagement [? ].

Online forums are a dynamic space where user behavior is constantly evolving. Given our data is from 2011-2015, we interpret the behavior in the context of standard online behavior at that time. Further research into the dynamics of online communities over the past decade would enrich the findings and conclusions of this study. Analyzing subsequent innovations in the Ultimaker community after 2015 could further validate the generalizability of the findings. Moreover, in this study user innovation is only explored in the context of hardware modifications and hacks. This is due to empirical data availability, where the user forum does not constitute a place for sharing software modifications. These limitations should caution wider generalizability. As a topic for future research, a broader exploration of user innovation in the Ultimaker product ecosystem could include software and firmware user-developer communities. Specific questions include whether there is an overlap between user innovators in hardware, and user-developers in software and firmware, as well as the extent to which these different communities exhibit different behavioral patterns. Further empirical study is needed to understand how user communities, user-producer communities, and the firm interact when it comes to software, hardware, and firmware innovation and integration, as these involve both digital and physical materiality [86] as well as different development processes and product interfaces. This is evidenced by the differing patterns of user contribution to firmware, versus hardware and software, in the Ultimaker user forum. Specific research questions include the extent to which physical materiality alters the nature and extent of user innovation in integrated physical-digital product ecosystems. Products such as 3D printers provide an opportunity to explore how the development and integration of both physical and digital goods components takes place in an open-source environment.

This paper supports the need for further research into the development of innovation behavior in user communities while also signalling the importance of advancing user innovation scholarship in the domain of open-source goods production [17]. More specifically, key questions remain on the relationship between user communities, user-producer communities, and the firm regarding product innovation in open-source physical goods. Greater understanding is needed in how the firm that produces the final product works with extended user-producer communities and their user community, and adopts product innovations advanced by both. In-depth community analysis could also reveal the role of embedded lead users [103] in both user-producer and user communities, the roles and behaviors of members that span these communities, and those that focus their time and attention in only one. Research is also needed into the motivations for occupying different community roles such as forum moderators, and the impact this may have on product innovation.

Finally, the empirical setting of open-source physical goods also opens possibilities of studying instances of user entrepreneurship, where companies are started by users of the product [104]. The question of how user entrepreneurship relates to the choice of an open source versus proprietary development path is interesting given the contrasting examples of Ultimaker and other 3D printer models on the market [89]. The question of whether user entrepreneurs choose to keep their product open, or whether they patent or restrict information for future product development, offers considerable prospects for furthering research into the costs and benefits of adopting open-source models of production.

## References

[1] von Hippel E. The dominant role of users in the scientific instrument innovation process. Res Policy 1976;5(3):212–39. doi: 10.1016/0048-7333(76)90028-7

[2] Rothwell R, Freeman C, Horlsey A, Jervis VTP, Robertson AB, Townsend J. SAPPHO updated - project SAPPHO phase II. Res Policy 1974;3(3):258–91. doi: 10.1016/0048-7333(74)90010-9

[3] von Hippel E. Lead users: a source of novel product concepts. Manage Sci 1986;32(7):791–805. doi: 10.1287/mnsc.32.7.791

[4] von Hippel E. The sources of innovation. New York, USA: Oxford University Press; 1988.

[5] Lettl C, Rost K, von Wartburg I. Why are some independent inventors “heroes” and others “hobbyists”? The moderating role of technological diversity and specialization. Res Policy 2009;38(2):243–54. doi: 10.1016/j.respol.2008.12.004

[6] Lüthje C, Herstatt C. The lead user method: an outline of empirical findings and issues for future research. R&D Manage 2004;34(5):553–68. doi: 10.1111/j.1467-9310.2004.00362.x

[7] Mahr D, Lievens A. Virtual lead user communities: drivers of knowledge creation for innovation. Res Policy 2012;41(1):167–77. doi: 10.1016/j.respol.2011.08.006

[8] Schreier M, Prügl R. Extending lead-user theory: antecedents and consequences of consumers’ lead userness. J Prod Innov Manage 2008;25(4):331–46. doi: 10.1111/j.1540-5885.2008.00305.x

[9] Jeppesen LB, Laursen K. The role of lead users in knowledge sharing. Res Policy 2009;38(10):1582–9. doi: 10.1016/j.respol.2009.09.002

[10] Schweisfurth TG. Comparing internal and external lead users as sources of innovation. Res Policy 2017;46(1):238–48. doi: 10.1016/j.respol.2016.11.002

[11] Magnusson PR. Exploring the contributions of involving ordinary users in ideation of technology-based services. J Prod Innov Manage 2009;26(5):578–93. doi: 10.1111/j.1540-5885.2009.00684.x

[12] Huang P, Tafti A, Mithas S. Platform sponsor investments and user contributions in knowledge communities: the role of knowledge seeding. MIS Q 2018;42(1):213–40. doi: 10.25300/MISQ/2018/13490

[13] Yan JK, Leidner DE, Benbya H. Differential innovativeness outcomes of user and employee participation in an online user innovation community. J Manag Inf Syst 2018;35(3):900–33. doi: 10.1080/07421222.2018.1481669

[14] Heiskanen E, Hyysalo S, Kotro T, Repo P. Constructing innovative users and user-inclusive innovation communities. Technol Anal Strateg Manag 2010;22(4):495–511. doi: 10.1080/09537321003714568

[15] Lüthje C, Herstatt C, von Hippel E. User-innovators and “local” information: The case of mountain biking. Res Policy 2005;34(6):951–65. doi: 10.1016/j.respol.2005.05.005

[16] von Hippel E, von Krogh G. Open Source software and the “private-collective” innovation model: issues for organization science. Organ Sci 2003;14(2):209–23. 10.2139/ssrn.1410789

[17] Benkler Y. When von Hippel innovation met the networked environment: recognizing decentralized innovation. In: Harhoff D, Lakhani KR, editors. Revolutionizing innovation: users, communities, and open innovation. Cambridge, MA: MIT Press; 2016. pp. 195–214.

[18] Raasch C, Herstatt C, Balka K. On the open design of tangible goods. R&D Manag 2009;39(4):382–93. 10.1111/j.1467-9310.2009.00567.x

[19] Benkler Y. Sharing nicely: On shareable goods and the emergence of sharing as a modality of economic production. The Yale Law Journal Company, Inc.; 2004. doi: 10.2307/4135731

[20] Bonvoisin J, Mies R, Boujut JF, Stark R. What is the “source” of open source hardware? J Open Hardw. 2017;1(1):1–18

[21] West J, Lakhani KR. Getting clear about communities in open innovation. Ind Innov. 2008;15(2):223–31. doi: 10.1080/13662710802033734

[22] Felin T, Zenger TR. Closed or open innovation? Problem solving and the governance choice. Res Policy 2014;43(5):914–25. doi: 10.1016/j.respol.2013.09.006

[23] Garmulewicz A. A dataset of contributions to the Ultimaker community forum 2011–2015. 2024. Available from: osf.io/bydma

[24] Flowers S. Harnessing the hackers: the emergence and exploitation of Outlaw Innovation. Res Policy 2008;37(2):177–93. doi: 10.1016/j.respol.2007.10.006

[25] Ultimaker. Episode 25: Ultimaker turns 10: a look back - Siert Wijnia, Ultimaker co-founder; 2021. Available from: https://www.talkingadditive.com/episodes/episode-25-ultimaker-turns-10-a-look-back

[26] Sheng ML, Chien I. Rethinking organizational learning orientation on radical and incremental innovation in high-tech firms. J Bus Res 2016;69(6):2302–8. doi: 10.1016/j.jbusres.2015.12.046

[27] Dewar R, Dutton J. The adoption of radical and incremental innovations : an empirical analysis. Manage Sci. 1986;32(11):1422–33. 10.1287/mnsc.32.11.1422

[28] Dutton J, Thomas A. Relating technological change and learning by doing. In: Rosenbloom R, editor. Research on technological innovation, management and policy. Jai Pr; 1985.

[29] Franke N, Shah S. How communities support innovative activities: an exploration of assistance and sharing among end-users. Res Policy 2003;32(1):157–78. 10.1016/S0048-7333(02)00006-9

[30] Raasch C, Herstatt C, Lock P. The dynamics of user innovation: drivers and impediments of innovation activities. Int J Innov Manage 2008;12(03):377–98. 10.1142/S1363919608002060

[31] Thomke S, Von Hippel E. Customers as innovators: a new way to create value. Harvard Business Review 2002;80.

[32] Harhoff D. Context, capabilities, and incentives—the core and the periphery of user innovation. In: Harhoff D, Lakhani KR, editors. Revolutionizing innovation: users, communities, and open innovation. Cambridge, MA: MIT Press; 2016. pp. 1–24.

[33] Lüthje C, Stockstrom C. Cost advantages in innovation—–a comparison of users and manufacturers. In: Harhoff D, Lakhani KR, editors. Revolutionizing innovation: users, communities, and open innovation. Cambridge, MA: MIT Press; 2016. pp. 45–65.

[34] Poetz MK, Schreier M. The value of crowdsourcing: can users really compete with professionals in generating new product ideas? J Product Innov Manage. 2012;29(2):245–56

[35] Kristensson P, Magnusson PR, Matthing J. Users as a hidden resource for creativity: findings from an experimental study on user involvement. Creat Innov Manag 2002;11(1):55–61. doi: 10.1111/1467-8691.00236

[36] Herstatt C, von Hippel E. From experience: developing new product concepts via the lead user method: a case study in a “low-tech” field. J Product Innov Manage 1992;9(3):213–21. doi: 10.1016/0737-6782(92)90031-7

[37] Hippel EV. The dominant role of the user in semiconductor and electronic subassembly process innovation. IEEE Trans Eng Manage. 1977;EM-24(2):60–71. doi: 10.1109/TEM.1977.6447336

[38] de Jong JPJ. The empirical scope of user innovation; 2016. In: Harhoff D, Lakhani K, editors. Revolutionizing innovation: users, communities and open innovation. MIT Press; 2016, pp. 67–87. doi: 10.7551/mitpress/9439.003.0007

[39] Liu Q, Du Q, Hong Y, Fan W, Wu S. User idea implementation in open innovation communities: evidence from a new product development crowd-sourcing community. Inf Syst J 2020;30(5):899–927. doi: 10.1111/isj.12286

[40] Oliveira P, von Hippel E. Users as service innovators: the case of banking services. Res Policy. 2011;40(6):806–18 doi: 10.1016/j.respol.2011.03.009

[41] Lakhani KR. Managing communities and contests to innovate with crowds. In: Harhoff D, Lakhani KR, editors. Revolutionizing innovation: users, communities, and open innovation. Cambridge, MA: The MIT Press; 2016. pp. 109–34. Available from: https://direct.mit.edu/books/book/4075/chapter/168976/managing-communities-and-contests-to-innovate-with

[42] Armstrong A, Hagel J. The real value of online communities. Harvard Bus Rev. 1996;74(3):134–41.

[43] Jeppesen LB, Frederiksen L. Why do users contribute to firm-hosted user communities? The case of computer-controlled music instruments. Organ Sci 2006;17(1):45–63. doi: 10.1287/orsc.1050.0156

[44] Parmentier G, Mangematin V. Orchestrating innovation with user communities in the creative industries. Technol Forecast Soc Change. 2014;83:40–53. doi: 10.1016/j.techfore.2013.03.007

[45] Costa J, Amorim I, Reis J, Melão N. User communities: from nice-to-have to must-have. J Innov Entrepreneurship 2023;12(1):25. doi: 10.1186/s13731-023-00292-1

[46] Faraj S, von Krogh G, Monteiro E, Lakhani KR. Special section introduction—online community as space for knowledge flows. Inf Syst Res 2016;27(4):668–84. doi: 10.1287/isre.2016.0682

[47] Yang M, Han C. Stimulating innovation: managing peer interaction for idea generation on digital innovation platforms. J Bus Res. 2021;125:456–65. doi: 10.1016/j.jbusres.2019.08.005

[48] Naik HS, Fritzsche A, Moeslein KM. Modularity in making: simplifying solution space for user innovation. R&D Manage 2021;51(1):57–72. doi: 10.1111/radm.12427

[49] Benkler Y. Sharing nicely: on shareable goods and the emergence of sharing as a modality of economic production. Yale Law J 2004;114(2):273–358. doi: 10.2307/4135731

[50] Di Gangi PM, Wasko MM, Hooker RE. Getting customers’ ideas to work for you: Learning from Dell how to succeed with online user innovation communities. MIS Q Exec. 2010;9(4):163–178.

[51] Jeppesen LB, Molin MJ. Consumers as co-developers: learning and innovation outside the firm. Technol Anal Strateg Manage 2003;15(3):363–83. doi: 10.1080/09537320310001601531

[52] Zhang J, Qi G, Wei K, Chen J. Spillover effects of interactions on user innovation: evidence from a firm-hosted open innovation platform. Inf Manage 2024;61(3):103947. doi: 10.1016/j.im.2024.103947

[53] Porter CE, Donthu N. Cultivating trust and harvesting value in virtual communities. Manag Sci 2008;54(1):113–28. doi: 10.1287/mnsc.1070.0765

[54] Franke N, Keinz P, Klausberger K. “Does this sound like a fair deal?”: antecedents and consequences of fairness expectations in the individual’s decision to participate in firm innovation. Organ Sci. 2012;24(5):1495–516. doi: 10.1287/orsc.1120.0794

[55] Füller J, Matzler K, Hoppe M. Brand community members as a source of innovation. J Prod Innov Manage 2008;25(6):608–619. doi: 10.1111/j.1540-5885.2008.00325.x

[56] Hienerth C, Lettl C, Keinz P. Synergies among producer firms, lead users, and user communities: the case of the LEGO producer–user ecosystem. J Prod Innov Manage 2014;31(4):848–66. doi: 10.1111/jpim.12127

[57] Li W, Lu Y, Ma J, Wang B. Users’ subsequent innovation after organizational adoption: evidence from an online game user innovation community. Internet Res 2023;33(4):1446–72. doi: 10.1108/INTR-08-2021-0578

[58] Liao X, Ye G, Yu J, Xi Y. Identifying lead users in online user innovation communities based on supernetwork. Ann Oper Res 2021;300(2):515–43. doi: 10.1007/s10479-021-03953-0

[59] Wang X, Ow TT, Liu L, Feng Y, Liang Y. Effects of peers and network position on user participation in a firm-hosted software community: the moderating role of network centrality. Eur J Inf Syst 2020;29(5):521–44. doi: 10.1080/0960085X.2020.1782275

[60] Mulhuijzen M, de Jong JPJ. Diffusion to peers in firm-hosted user innovation communities: contributions by professional versus amateur users. Res Policy 2024;53(1):104897. doi: 10.1016/j.respol.2023.104897

[61] Lüthje C. Characteristics of innovating users in a consumer goods field: an empirical study of sport-related product consumers. Technovation 2004;24(9):683–95. doi: 10.1016/S0166-4972(02)00150-5

[62] von Hippel E. Democratizing innovation. Cambridge, MA: MIT Press; 2005.

[63] Comino S, Manenti FM, Parisi ML. From planning to mature: on the success of open source projects. Res Policy 2007;36(10):1575–86. doi: 10.1016/j.respol.2007.08.003

[64] Raymond ES. The cathedral & the bazaar: musings on Linux and Open Source by an accidental revolutionary. Sebastopol, CA: O’Reilly Media; 2001.

[65] Lerner J, Tirole J. Some simple economics of Open Source. J Ind Econ 2002;50(2):197–234. doi: 10.1111/1467-6451.00174

[66] David PA, Shapiro JS. Community-based production of Open Source software: what do we know about the developers who participate? Inf Econ Pol. 2008;20(4):364–98

[67] Faraj S, Jarvenpaa SL, Majchrzak A. Knowledge collaboration in online communities. Organization Science. 2011;22(5):1224–1239. doi: 10.1287/orsc.1100.0614

[68] von Krogh G, von Hippel E. The promise of research on Open Source software. Manage Sci 2006;52(7):975–83. doi: 10.1287/mnsc.1060.0560

[69] Moon JY, Sproull L. Essence of distributed work: the case of the Linux kernel. First Monday. 2005;5(11). doi: 10.5210/fm.v0i0.1479

[70] Hertel G, Niedner S, Herrmann S. Motivation of software developers in Open Source projects: an Internet-based survey of contributors to the Linux kernel. Res Policy 2003;32(7):1159–77. doi: 10.1016/S0048-7333(03)00047-7

[71] Bitzer J, Schrettl W, Schröder PJH. Intrinsic motivation in open source software development. J Comp Econ 2007;35(1):160–9. doi: 10.1016/j.jce.2006.10.001

[72] Shah SK. Motivation, governance, and the viability of hybrid forms in Open Source software development. Manage Sci 2006;52(7):1000–14. doi: 10.2139/ssrn.898247

[73] Roberts JA, Hann IH, Slaughter SA. Understanding the motivations, participation, and performance of Open Source software developers: a longitudinal study of the Apache projects. Manage Sci. 2006;52(7):984–99. doi: 10.1287/mnsc.1060.0554

[74] Benkler Y. Coase’s Penguin, or, Linux and the nature of the firm. Yale Law J 2002;112(3):369. doi: 10.2307/1562247

[75] Benkler Y. Intellectual property: commons-based strategies and the problems of patents. Science 2004;305(5687):1110–1. doi: 10.1126/science.1100526 15326340

[76] Baldwin CY, Clark KB. The architecture of participation: does code architecture mitigate free riding in the open source development model? Manage Sci. 2006;52(7):1116–27

[77] Grewal R, Lilien GL, Mallapragada G. Location, location, location: how network embeddedness affects project success in Open Source systems. Manage Sci 2006;52(7):1043–56. doi: 10.1287/mnsc.1060.0550

[78] O’Mahony S. Guarding the commons: how community managed software projects protect their work. Res Policy 2003;32(7):1179–1198. doi: 10.1016/S0048-7333(03)00048-9

[79] Mellis D, Buechley L. Collaboration in Open-source hardware: third-party variations on the Arduino Duemilanove. In: Proceedings of the ACM 2012 Conference on Computer Supported Cooperative Work. CSCW ’12. New York, NY, USA: ACM; 2012. pp. 1175–8. Available from: http://doi.acm.org/10.1145/2145204.2145377

[80] Kuznetsov S, Doonan C, Wilson N, Mohan S, Hudson SE, Paulos E. DIYbio things: Open Source biology tools as platforms for hybrid knowledge production and scientific participation. In: Proceedings of the 33rd Annual ACM Conference on Human Factors in Computing Systems. CHI ’15. New York, NY, USA: ACM; 2015. pp. 4065–8. Available from: http://doi.acm.org/10.1145/2702123.2702235

[81] Baden T, Chagas AM, Gage G, Marzullo T, Prieto-Godino LL, Euler T. Open Labware: 3-D printing your own lab equipment. PLOS Biol. 2015;13(3):1–12. doi: 10.1371/journal.pbio.1002086 PMC4368627 25794301

[82] Anzalone GC, Glover AG, Pearce JM. Open-Source colorimeter. Sensors 2013;13(4):5338–46. doi: 10.3390/s130405338 23604032 PMC3673140

[83] Wittbrodt BT, Glover AG, Laureto J, Anzalone GC, Oppliger D, Irwin JL, et al. Life-cycle economic analysis of distributed manufacturing with open-source 3-D printers. Mechatronics 2013;23(6):713–26. doi: 10.1016/j.mechatronics.2013.06.002

[84] Anzalone GC, Zhang C, Wijnen B, Sanders PG, Pearce JM. A low-cost Open-Source metal 3-D printer. IEEE Access. 2013;1:803–10. doi: 10.1109/ACCESS.2013.2293018

[85] Pearce JM. Building research equipment with free, open-source hardware. Science. 2012;337(6100):1303–4 doi: 10.1126/science.1228183 22984059

[86] Yoo Y, Boland RJ, Lyytinen K, Majchrzak A. Organizing for innovation in the digitized world. Organ Sci. 2012;23(5):1398–408. doi: 10.1287/orsc.1120.0771

[87] Von Hippel E. Innovation by user communities: learning from Open-Source software. MIT Sloan Manage Rev. 2001;42(4):82–6.

[88] Raasch C. Product development in open design communities: a process perspective. Int J Innov Technol Manage 2011;08(04):557–75. doi: 10.1142/S021987701100260X

[89] West J, Kuk G. The complementarity of openness: how MakerBot leveraged thingiverse in 3D printing. Technol Forecast Soc Change. 2016;102:169–81. doi: 10.1016/J.TECHFORE.2015.07.025

[90] Özkil AG. Collective design in 3D printing:a large scale empirical study of designs, designers and evolution. Design Stud. 2017;51:66–89. doi: 10.1016/J.DESTUD.2017.04.004

[91] Flath CM, Friesike S, Wirth M, Thiesse F. Copy, transform, combine: exploring the remix as a form of innovation. J Inf Technol 2017;32(4):306–25. doi: 10.1057/s41265-017-0043-9

[92] Friesike S, Flath CM, Wirth M, Thiesse F. Creativity and productivity in product design for additive manufacturing: mechanisms and platform outcomes of remixing. J Oper Manag 2019;65(8):735–52. doi: 10.1016/j.jom.2018.10.004

[93] Claussen J, Halbinger MA. The role of pre-innovation platform activity for diffusion success:evidence from consumer innovations on a 3D printing platform. Res Policy 2021;50(8):103943. doi: 10.1016/j.respol.2020.103943.

[94] Troxler P, Wolf P. Digital maker-entrepreneurs in open design: what activities make up their business model? Bus Horiz. 2017;60(6):807–17

[95] Molitch-Hou M. Has MakerBot become TakerBot? - 3D printing industry; 2014. Available from: http://3dprintingindustry.com/2014/05/28/makerbot-become-takerbot/

[96] Balka K, Raasch C, Herstatt C. The effect of selective openness on value creation in user innovation communities. J Prod Innov Manage 2014;31(2):392–407. doi: 10.1111/jpim.12102

[97] Lozova L. Company update: Ultimaker files first patents; 2017. Available from: https://ultimaker.com/learn/company-update-ultimaker-files-first-patents

[98] Harhoff D. Context, capabilities, and incentives—the core and the periphery of user innovation. In: Harhoff D, Lakhani KR, editors. Revolutionizing innovation: users, communities, and open innovation. Cambridge, MA: MIT Press; 2016. pp. 27–44.

[99] Wasserman S, Faust K. Models and methods in social network analysis. Cambridge, UK: Cambridge University Press; 1994.

[100] von Krogh G, Spaeth S, Lakhani KR. Community, joining, and specialization in open source software innovation: a case study. Res Policy 2003;32(7):1217–41. doi: 10.1016/S0048-7333(03)00050-7

[101] Peuckert J, Kern F. How user innovation communities contribute to sustainability transitions. An exploration of three online communities. Environ Innov Soc Transit. 2023;49:100785. doi: 10.1016/j.eist.2023.100785

[102] Snijders TA, Bosker RJ. Multilevel analysis: an introduction to basic and advanced multilevel modeling, 2nd edn. London, UK: Sage Publications; 2012.

[103] Herstatt C, Schweisfurth T, Raasch C. When passion meets profession: how embedded lead users contribute to corporate innovation. In: Harhoff D, Lakhani KR, editors. Revolutionizing innovation: users, communities, and open innovation. Cambridge, MA: MIT Press; 2016. pp. 397–420.

[104] Shah SK, Tripsas M. When do user-innovators start firms? A theory of user entrepreneurship. In: Harhoff D, Lakhani KR, editors. Revolutionizing innovation: users, communities, and open innovation. Cambridge, MA: MIT Press; 2016. pp. 285–308.

